# Complementary Chinese Herbal Medicine Therapy Improves Survival in Patients With Pemphigus: A Retrospective Study From a Taiwan-Based Registry

**DOI:** 10.3389/fphar.2020.594486

**Published:** 2020-12-09

**Authors:** Po-Yuan Wu, Te-Mao Li, Shu-I. Chen, Chao-Jung Chen, Jian-Shiun Chiou, Ming-Kuem Lin, Fuu-Jen Tsai, Yang-Chang Wu, Ting-Hsu Lin, Chiu-Chu Liao, Shao-Mei Huang, Yu-Ning Lin, Wen-Miin Liang, Ying-Ju Lin

**Affiliations:** ^1^ Department of Dermatology, China Medical University Hospital, Taichung, Taiwan; ^2^ School of Medicine, China Medical University, Taichung, Taiwan; ^3^ School of Chinese Medicine, China Medical University, Taichung, Taiwan; ^4^ Department of Chinese Medicine, Asia University Hospital, Taichung, Taiwan; ^5^ Proteomics Core Laboratory, Genetic Center, Department of Medical Research, China Medical University Hospital, Taichung, Taiwan; ^6^ Graduate Institute of Integrated Medicine, China Medical University, Taichung, Taiwan; ^7^ Department of Health Services Administration, China Medical University, Taichung, Taiwan; ^8^ Department of Chinese Pharmaceutical Sciences and Chinese Medicine Resources, China Medical University, Taichung, Taiwan; ^9^ Department of Biotechnology and Bioinformatics, Asia University, Taichung, Taiwan

**Keywords:** pemphigus, overall mortality, Chinese herbal medicine, association rule mining, network

## Abstract

Pemphigus is a life-threatening and skin-specific inflammatory autoimmune disease, characterized by intraepidermal blistering between the mucous membranes and skin. Chinese herbal medicine (CHM) has been used as an adjunct therapy for treating many diseases, including pemphigus. However, there are still limited studies in effects of CHM treatment in pemphigus, especially in Taiwan. To more comprehensively explore the effect of long-term CHM treatment on the overall mortality of pemphigus patients, we performed a retrospective analysis of 1,037 pemphigus patients identified from the Registry for Catastrophic Illness Patients database in Taiwan. Among them, 229 and 177 patients were defined as CHM users and non-users, respectively. CHM users were young, predominantly female, and had a lesser Charlson comorbidity index (CCI) than non-CHM users. After adjusting for age, sex, prednisolone use, and CCI, CHM users had a lower overall mortality risk than non-CHM users (multivariate model: hazard ratio (HR): 0.422, 95% confidence interval (CI): 0.242–0.735, *p* = 0.0023). The cumulative incidence of overall survival was significantly higher in CHM users than in non-users (*p* = 0.0025, log rank test). Association rule mining and network analysis showed that there was one main CHM cluster with Qi–Ju–Di–Huang–Wan (QJDHW), Dan–Shen (DanS; *Radix Salviae miltiorrhizae*; *Salvia miltiorrhiza Bunge*), Jia–Wei–Xiao–Yao-–San (JWXYS), Huang–Lian (HL; *Rhizoma coptidis*; *Coptis chinensis Franch*.), and Di–Gu–Pi (DGP; *Cortex lycii*; *Lycium barbarum L.*), while the second CHM cluster included Jin–Yin–Hua (JYH; *Flos lonicerae*; *Lonicera hypoglauca Miq.*) and Lian–Qiao (LQ; *Fructus forsythiae*; *Forsythia suspensa (Thunb.) Vahl*). In Taiwan, CHMs used as an adjunctive therapy reduced the overall mortality to approximately 20% among pemphigus patients after a follow-up of more than 6 years. A comprehensive CHM list may be useful in future clinical trials and further scientific investigations to improve the overall survival in these patients.

## Introduction

Pemphigus is a life-threatening and skin-specific inflammatory autoimmune disease, characterized by painful erosion of mucous membranes or flaccid blistering of the skin, in addition to mucous membrane lesions (Didona et al., 2019). This intraepidermal blistering is called acantholysis due to the loss of cell-cell adhesion, and is predominantly induced by pathogenic IgG autoantibodies (autoAbs) against epidermal adhesion proteins of keratinocytes (Buonavoglia et al., 2019). Pemphigus includes two major types: pemphigus vulgaris (PV) and pemphigus foliaceus (PF). Other types with varying degrees of inflammation and IgG autoAb concentrations against adhesion proteins have also been reported (Amber et al., 2018; Chernyavsky et al., 2019). Patients with PV possess IgG auto Abs mainly against desmoglein 3 (anti-Dsg3 autoAbs) or anti-Dsg3 and anti-Dsg1 autoAbs (Sinha and Sajda, 2018; Walter et al., 2019), while patients with PF have anti-Dsg 1 autoAbs (Russo et al., 2017; Oktarina et al., 2019). Patients with other types have a variety of IgG autoAbs against the plakin family, plakophilin 3, desmocollins 1 and 3, and alpha-2 macroglobulin-like 1 adhesion proteins (Kasperkiewicz et al., 2017; Kim and Kim, 2019).

Before the usage of systemic prednisolone, pemphigus vulgaris was considered a fatal disease, with most patients dying within 2–5 years after disease onset (Porro et al., 2019). Glucocorticoid treatment generally controls the acute stage of pemphigus and its prolonged use has been the standard of care for this disease (Gregoriou et al., 2015; Didona et al., 2019). The estimated mortality rate remains 2.36 times higher compared to standard mortality rate after the use of systemic steroids in Taiwan (Huang et al., 2012). Adverse effects of glucocorticoid treatment in pemphigus have also been reported, including the development of diabetes mellitus, osteoporosis, hypertension, insomnia, and gastrointestinal problems (Cholera and Chainani-Wu, 2016). Novel therapies for pemphigus have been developed using immunosuppressive agents that target specific proteins in B cells to prevent pathogenic autoantibody production (Musette and Bouaziz, 2018; Bilgic and Murrell, 2019; Didona et al., 2019; Izumi et al., 2019). Currently, the target specific proteins include CD20, CD19, and Bruton’s tyrosine kinase (BTK) (Bilgic and Murrell, 2019; Didona et al., 2019; Dhillon, 2020; Goodale et al., 2020a; Goodale et al., 2020b). The B cell inhibitors which block CD20 include rituximab, veltuzumab, ofatumumab, have been developed in clinical trials (Didona et al., 2019). The CD19 inhibitors include inebilizumab (Didona et al., 2019). The BTK inhibitors include PRN1008, tirabrutinib, and PRN-473 (Dhillon, 2020; Goodale et al., 2020a; Goodale et al., 2020b). However, adverse effects from prolonged use of these novel immunosuppressive agents remain to be elucidated.

Chinese herbal medicine (CHM) has served as an adjunctive therapy in many diseases, and has been an important aspect of the Taiwan health care system since 1995 (Lin et al., 2015b; Tsai et al., 2017a; Li et al., 2018; Tsai et al., 2018; Cheng et al., 2019; Tsai et al., 2019a; Tsai et al., 2019b). Furthermore, CHM has been used extensively to treat skin diseases in Taiwan, including psoriasis, eczema, atopic dermatitis, and pemphigus (Arichi et al., 1979; Lin et al., 2014; Chen et al., 2015; Zhou et al., 2015b; Weng et al., 2016). However, there are still limited studies in effects of CHM treatment in pemphigus, especially in Taiwan. Furthermore, the beneficial effects and safety of glucocorticoid treatment combined with CHM have been observed in patients with pemphigus (Arichi et al., 1979; Zhou et al., 2015b).

To explore the prolonged effect of CHM treatment on patients with pemphigus, we utilized a population-based database to investigate demographic characteristics, cumulative incidence of overall mortality, and CHM prescription patterns. Through this nation-wide population-based analysis in Taiwan, we aim to investigate whether the use of CHM as adjunctive therapy offered benefits to patients with pemphigus.

## Materials and Methods

### Study Subjects

This study was approved by the Research Ethics Committee of the Taiwan National Health Research Institute and the Institutional Review Board of the China Medical University Hospital (ethics approval number: CMUH107-REC3-074(CR1)). The research was a longitudinal and retrospective cohort study, carried out between 2000 and 2016, using the database of Registry for Catastrophic Illness Patients of Taiwan’s National Health Insurance Research Database (NHIRD; http://nhird.nhri.org.tw/) from the National Health Insurance (NHI) program in Taiwan.

**FIGURE 1:**
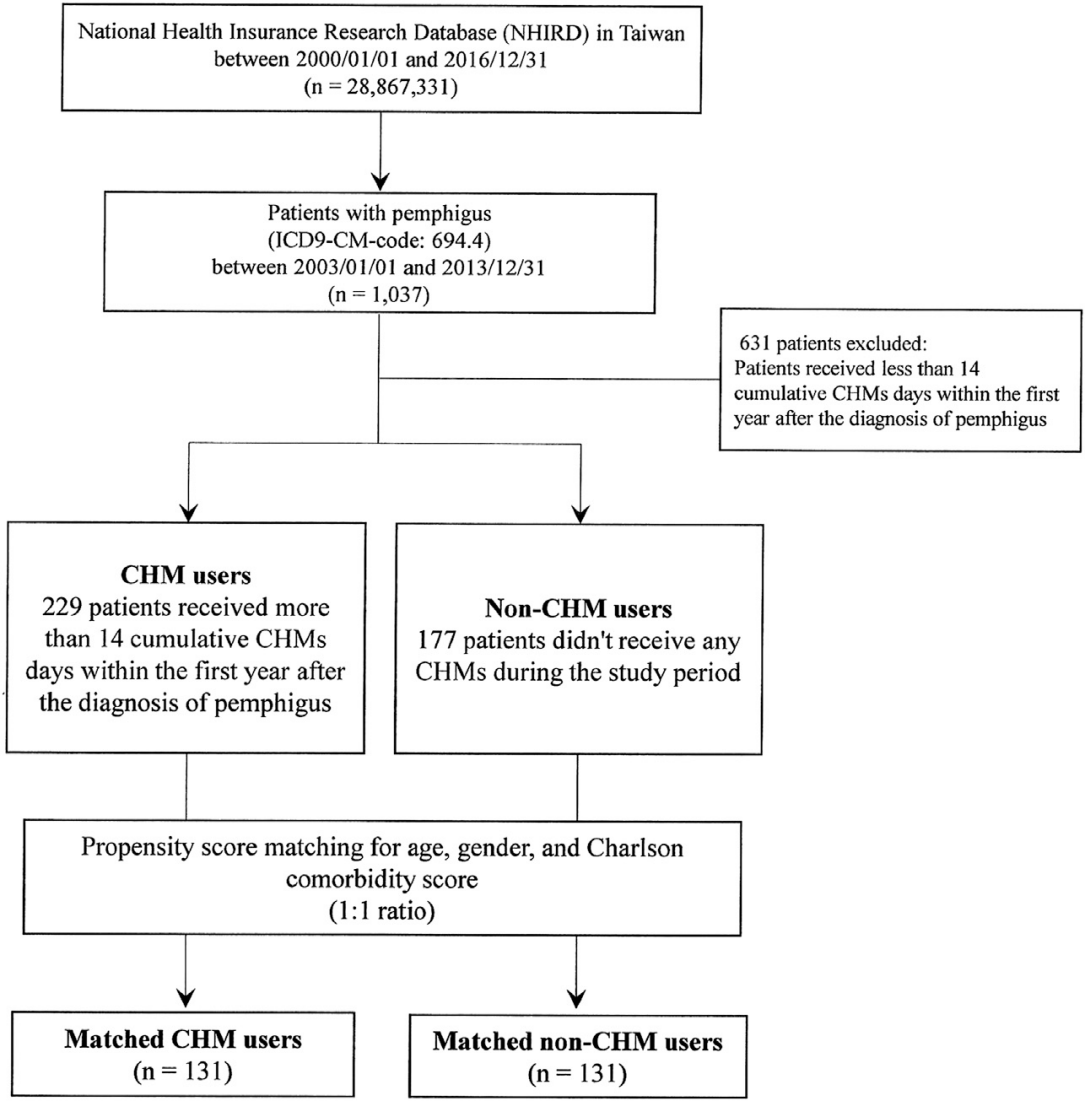
Flowchart for the enrollment of CHM and non-CHM users in patients with pemphigus. Abbreviations: CHM, Chinese herbal medicine.

In this study, all the patients were deidentified, and patients with pemphigus were identified using the International Classification of Disease, 9th Revision, Clinical Modification (ICD-9-CM). A total of 1,037 patients with pemphigus (ICD-9-CM-code: 694.4) were identified during the 2003–2013 period (Figure 1). Patients who received less than 14 cumulative CHMs days within the first year after the diagnosis of pemphigus were excluded (*n* = 631), and patients were defined as CHM users when they had more than 14 cumulative CHM treatment days within the first year after the diagnosis of pemphigus (*n* = 229, Figure 1). The index date started on the day wherein the 14 cumulative days of CHM treatment had been completed. The CHM users continued to use CHMs during the study period (from the index date to the study endpoint). Patients were defined as non-CHM users if they did not receive any CHMs during the study period (*n* = 177). The study endpoint for overall mortality was defined as the date of withdrawal from the NHI program or the date of termination of follow-up (December 31, 2016).

There were 229 and 177 patients classified as CHM users and non-users, respectively (Table 1). Propensity score matching was applied to match the CHM and non-CHM users for age, gender, and Charlson comorbidity score (1:1 ratio). After matching, there were 131 matched CHM and 131 non-CHM users, respectively (Table 1). The Charlson comorbidity index (CCI) and Charlson comorbidities were identified before the patients’ diagnosis with pemphigus. The Charlson comorbidities included congestive heart failure (ICD-9-CM: 398.91, 402.01, 402.11, 402.91, 404.01, 404.03, 404.11, 404.13, 404.91, 404.93, 425.4–425.9, 428.x), peripheral vascular disease (ICD-9-CM: 093.0, 437.3, 440.x, 441.x, 443.1–443.9, 447.1, 557.1, 557.9, V43.4), cerebrovascular disease (ICD-9-CM: 362.34, 430.x–438.x), chronic pulmonary disease (ICD-9-CM: 416.8, 416.9, 490.x–505.x, 506.4, 508.1, 508.8), rheumatic disease (ICD-9-CM: 446.5, 710.0–710.4, 714.0–714.2, 714.8, 725.x), peptic ulcer disease (ICD-9-CM: 531.x - 534.x), mild liver disease (ICD-9-CM: 070.22, 070.23, 070.32, 070.33, 070.44, 070.54, 070.6, 070.9, 570.x, 571.x, 573.3, 573.4, 573.8, 573.9, V42.7), diabetes without chronic complications (ICD-9-CM: 250.0–250.3, 250.8, 250.9), diabetes with chronic complications (ICD-9-CM: 250.4–250.7), hemiplegia or paraplegia (ICD-9-CM: 334.1, 342.x, 343.x, 344.0–344.6, 344.9), renal disease (ICD-9-CM: 403.01, 403.11, 403.91, 404.02, 404.03, 404.12, 404.13, 404.92, 404.93, 582.x, 583.0–583.7, 585.x, 586.x, 588.0, V42.0, V45.1, V56.x), and any malignancy, including lymphoma and leukemia, except malignant neoplasms of the skin (ICD-9-CM: 140.x–172.x, 174.x–195.8, 200.x–208.x, 238.6) (Table 1).

**TABLE 1:** Demographic characteristics between CHM and non-CHM groups among patients with pemphigus in Taiwan.

Characteristics	Total patients		*p*-value	Matched patients		*p*-value
	CHM group (*N* = 229)	Non-CHM group (*N* = 177)		CHM group (*N* = 131)	Non-CHM group (*N* = 131)	
	*N* (%)	*N* (%)		*N* (%)	*N* (%)	
Age (Mean ± SD)						
0 ≦ Age < 40	55 (24.02%)	23 (12.99%)	***<0.001***	22 (16.79%)	23 (17.56%)	0.875
40 ≦ Age < 50	66 (28.82%)	28 (15.82%)		29 (22.14%)	28 (21.37%)	
50 ≦ Age < 60	58 (25.33%)	29 (16.38%)		34 (25.95%)	29 (22.14%)	
60 ≦ Age	50 (21.83%)	97 (54.8%)		46 (35.11%)	51 (38.93%)	
Gender			***<0.001***			0.531
Male	97 (42.54%)	114 (64.41%)		79 (60.31%)	74 (56.49%)	
Female	131 (57.46%)	63 (35.59%)		52 (39.69%)	57 (43.51%)	
Prednisolone use			0.192			0.452
No	13 (5.68%)	16 (9.04%)		7 (5.34%)	10 (7.63%)	
Yes	216 (94.32%)	161 (90.96%)		124 (94.66%)	121 (92.37%)	
Charlson comorbidity index (CCI) score (Mean ± SD)	1.16 ± 1.56	1.79 ± 2.33	***0.002***	1.46 ± 1.77	1.54 ± 2.3	0.741

p-values were obtained by chi-square test. *p*-value (*p* < 0.05) was highlighted in bold italic.

CHM, Chinese herbal medicine; N, number; CCI, Charlson comorbidity index.

Notes: Pemphigus (ICD-9-CM-code: 694.4). The Charlson comorbidities include congestive heart failure (ICD-9-CM: 398.91, 402.01, 402.11, 402.91, 404.01, 404.03, 404.11, 404.13, 404.91, 404.93, 425.4–425.9, 428.x), peripheral vascular disease (ICD-9-CM: 093.0, 437.3, 440.x, 441.x, 443.1–443.9, 447.1, 557.1, 557.9, V43.4), cerebrovascular disease (ICD-9-CM: 362.34, 430.x–438.x), chronic pulmonary disease (ICD-9-CM: 416.8, 416.9, 490.x–505.x, 506.4, 508.1, 508.8), rheumatic disease (ICD-9-CM: 446.5, 710.0–710.4, 714.0–714.2, 714.8, 725.x), peptic ulcer disease (ICD-9-CM: 531.x–534.x), mild liver disease (ICD-9-CM: 070.22, 070.23, 070.32, 070.33, 070.44, 070.54, 070.6, 070.9, 570.x, 571.x, 573.3, 573.4, 573.8, 573.9, V42.7), diabetes without chronic complication (ICD-9-CM: 250.0–250.3, 250.8, 250.9), diabetes with chronic complication (ICD-9-CM: 250.4–250.7), hemiplegia or paraplegia (ICD-9-CM: 334.1, 342.x, 343.x, 344.0–344.6, 344.9), renal disease (ICD-9-CM: 403.01, 403.11, 403.91, 404.02, 404.03, 404.12, 404.13, 404.92, 404.93, 582.x, 583.0–583.7, 585.x, 586.x, 588.0, V42.0, V45.1, V56.x), and any malignancy, including lymphoma and leukemia, except malignant neoplasm of skin (ICD-9-CM: 140.x–172.x, 174.x–195.8, 200.x–208.x, 238.6). These comorbidities were recorded before the diagnosis of pemphigus. Propensity score matching was performed for age, gender, and CCI score (1:1 ratio).

### Chinese Herbal Medicine

Chinese Herbal Medicine (CHM) contains two types- single herb and herbal formulas and is prescribed by licensed and experienced traditional Chinese medicine (TCM) doctors in Taiwan (https://www.nhi.gov.tw/Content_List.aspx?n=A068D27CBF677629&topn=5FE8C9FEAE863B46&upn=7A70FD46553E5155). A single herb may be made from the flower, root, stem, or leaf of a plant. Single herbs may also be made from the organs of animals, insects, or mineral sources. The herbal formulas are mixtures of a minimum of two single herbs. In this study, the CHM composition, dosage, frequency, and usage patterns for patients with pemphigus are shown in Supplementary Table S1. The CHM products used in this study are all produced by pharmaceutical manufacturers with Good Manufacturing Practice in Taiwan. These pharmaceutical manufacturers include Sun Ten Pharmaceutical Co. Ltd. (http://www.sunten.com.tw/), Chuang Song Zong Pharmaceutical Co. Ltd. (http://www.csz.com.tw/), Shang Chang Pharmaceutical Co. Ltd. (http://www.herb.com.tw/about_en.php), KO DA Pharmaceutical Co. Ltd. (http://www.koda.com.tw/), and Kaiser Pharmaceutical Co. Ltd. (http://www.kpc.com/).

### Association Rule and Network Analysis

The association rule method for two-CHM combinations (Table 3) was described and performed as in previous studies (Cheng et al., 2019; Tsai et al., 2019a; Tsai et al., 2019b), and computed using the SAS software (version 9.4; SAS Institute, Cary, NC, United States). The support value (%) is the calculated joint probability of having both prescriptions of CHM_X and CHM_Y products, i.e. frequency of prescriptions of CHM_X and CHM_Y products/total prescriptions × 100%. The confidence value (CHM_X → CHM_Y; %) is the calculated conditional probability of having prescription of CHM_Y among those who already have the prescription of CHM_X, i.e., frequency of prescriptions of CHM_X and CHM_Y products/frequency of prescription of CHM_X product × 100%. The lift value is confidence (CHM_X →CHM_Y) (%)/P (Y) (%) or confidence (CHM_Y → CHM_X) (%)/P (X) (%), both with the same value in the mathematical formula.

Cytoscape (https://cytoscape.org/, version 3.7.0) was used to analyze the network for CHM clusters for patients with pemphigus (Figure 3). Network analysis was also described and performed in previous studies (Cheng et al., 2019; Tsai et al., 2019a; Tsai et al., 2019b). The lines connecting CHMs represent the support value, with thicker lines representing higher support values. The line color between CHMs shows the lift value, with darker lines representing stronger connections with higher lift values. The red circle represents herbal formulas while the green circle represents single herbs. The size of the circle for each CHM shows its own prescription frequency, with larger circles indicating higher prescription frequency.

### Statistical Analysis

SAS software (version 9.4; SAS Institute, Cary, NC, United States) was used for data management and statistical analyses. Categorical data, including age, gender, and prednisolone use, were expressed in numbers and percentages (Table 1). The significance of the differences in categorical data were calculated using Chi-squared tests. Charlson comorbidity index (CCI) scores were expressed as continuous data (mean ± SD; Table 1). The significance of the difference in continuous data were calculated using Student’s t tests.

**FIGURE 2:**
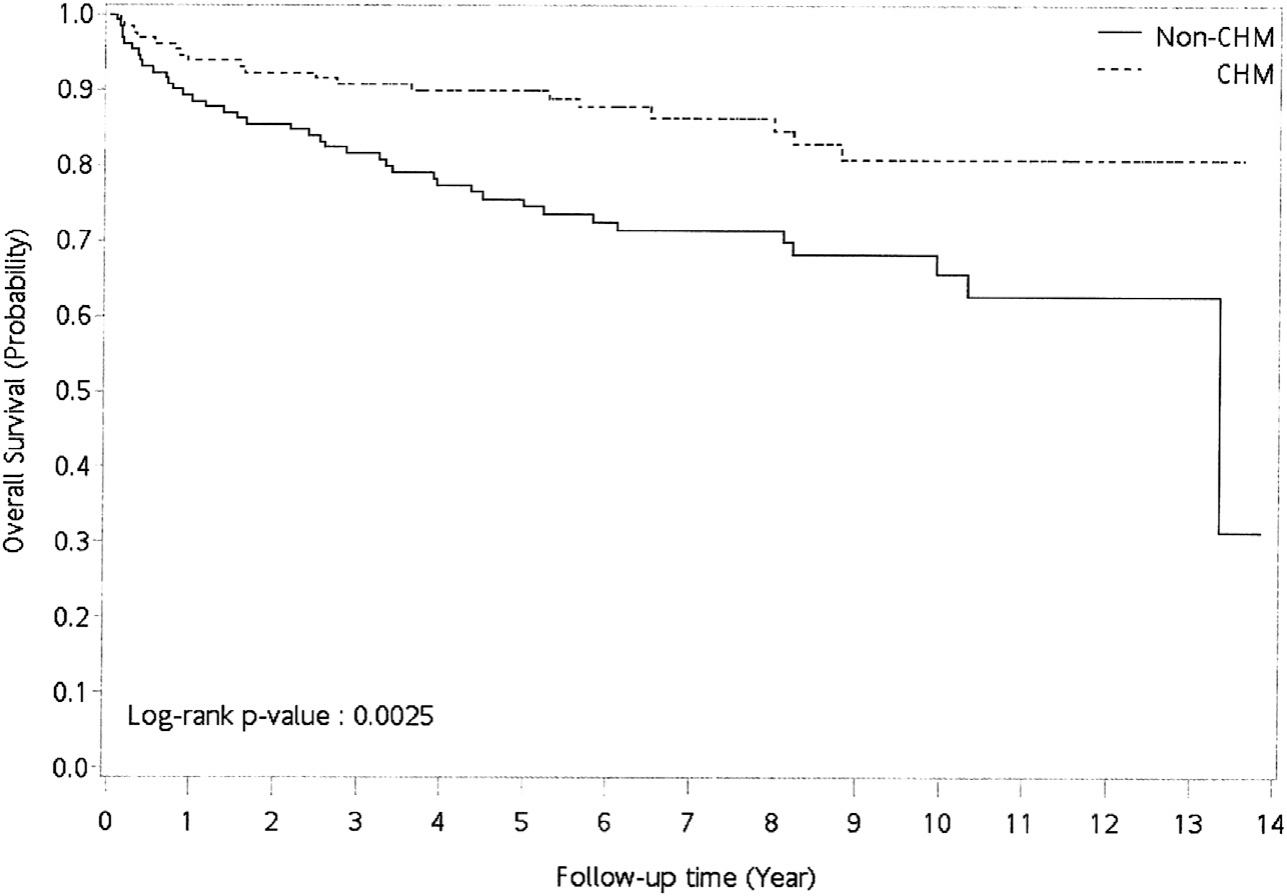
Cumulative incidence of overall survival between CHM and non-CHM users in patients with pemphigus. Abbreviations: CHM, Chinese herbal medicine.

A univariate Cox proportional hazard model was performed to evaluate the hazard ratio (HR) and 95% confidence interval (CI) of risk of overall mortality between CHM and non-users (Table 2). The multivariate Cox proportional hazard model was also performed by adjusting for age, gender, prednisolone use, CCI score, and CHM use (Table 2). The Kaplan–Meier survival plot and the log-rank test were applied to assess the 14-years cumulative incidence of overall survival between CHM and non-CHM users (Figure 2). All *p*-values less than 0.05 were considered as statistically significant differences.

**TABLE 2:** Cox proportional hazard models for overall mortality in patients with pemphigus in Taiwan.

	Univariate			Multivariate		
	HR	95% CI	*p*-value	aHR	95% CI	*p*-value
Age						
0 ≦ Age < 40	Ref.	ND	ND	Ref.	ND	ND
40 ≦ Age < 50	0.486	(0.08–2.91)	0.4294	0.300	(0.048–1.865)	0.1967
50 ≦ Age < 60	2.305	(0.62–8.53)	0.2112	1.853	(0.498–6.888)	0.3572
60 ≦ Age	8.838	(2.74–28.47)	***<0.0001***	5.229	(1.535–17.81)	***0.0082***
Gender						
Male	Ref.	ND	ND	Ref.	ND	ND
Female	1.213	(0.73–2.03)	0.461	0.931	(0.545–1.592)	0.794
Prednisolone use						
No	Ref.	ND	ND	Ref.	ND	ND
Yes	1.551	(0.483–4.975)	0.4607	2.775	(0.757–10.172)	0.1235
Charlson comorbidity index (CCI) score, per score	1.385	(1.28–1.5)	***<0.0001***	1.278	(1.156–1.412)	***<0.0001***
CHM use						
No	Ref.	ND	ND	Ref.	ND	ND
Yes	0.441	(0.26–0.76)	***0.0033***	0.422	(0.242–0.735)	***0.0023***

CHM, Chinese herbal medicine; CCI, Charlson comorbidity index; HR, hazard ratio; aHR, adjusted hazard ratio; CI, confidence interval; Ref., reference; ND, not determined.

Notes: Pemphigus (ICD-9-CM-code: 694.4). Prednisolone use was presented before their diagnosis of pemphigus. The multivariate Cox proportional hazard model was performed by adjusting for age, gender, prednisolone use, CCI score, and CHM use. *p*-value (*p* < 0.05) was shown in bold italic font.

## Results

### Basic Characteristics of Patients With Pemphigus in Taiwan

Patients with pemphigus (ICD-9-CM-code: 694.4) in Taiwan were selected from the database of Registry for Catastrophic Illness Patients of Taiwan’s National Health Insurance Research Database (NHIRD) (Figure 1). As shown, there were 1,037 patients with pemphigus identified during the period between January 1, 2003 and December 31, 2013. After excluding patients who received less than 14 cumulative Chinese herbal medicine (CHM) days within the first year after the diagnosis of pemphigus, there were 229 patients classified as CHM users and 177 patients as non-CHM users. CHM users were defined as patients who received more than 14 cumulative CHM days within the first year after the diagnosis of pemphigus, while non-CHM users were defined as patients who did not receive any CHMs during the study period (Figure 1). There were significant differences in age, gender, and Charlson comorbidity scores between CHM and non-CHM users (*p*-value < 0.05; Table 1); with CHM users being younger, predominantly female, and having lower Charlson comorbidity scores compared to non-users. To eliminate any potential confounding factors, propensity score matching was applied to match the CHM and non-CHM users for age, gender, and Charlson comorbidity scores (1:1 ratio). After matching, there were similar demographic characteristics between the matched CHM and non-CHM users (*p*-value > 0.05; Table 1).

### Risk of Overall Mortality in Patients With Pemphigus in Taiwan

The risk of overall mortality in patients with pemphigus was investigated using univariate and multivariate Cox proportional hazard models (Table 2). There were significant differences in age, Charlson comorbidity index (CCI), and Chinese herbal medicine (CHM) usage. Univariate and multivariate Cox proportional hazard models showed that patients over 60 years old had a higher risk of overall mortality than those below 40 years old (Table 2; univariate model: hazard ratio (HR): 8.838, 95% confidence interval (CI): 2.74–28.47, *p* < 0.0001; multivariate model: HR: 5.229, 95% CI: 1.535–17.81, *p* = 0.0082). Patients had a higher risk of overall mortality when they had every point increase in their Charlson comorbidity index (CCI) score (Table 2; univariate model: HR: 1.385, 95% CI: 1.28–1.50, *p* < 0.0001; multivariate model: HR: 1.278, 95% CI: 1.156–1.412, *p* < 0.0001).

Patients who used CHM had a lower risk of overall mortality than non-CHM users (Table 2; univariate model: HR: 0.441, 95% CI: 0.26–0.76, *p* = 0.0033; multivariate model: HR: 0.422, 95% CI: 0.242–0.735, *p* = 0.0023). Kaplan–Meier survival plots also showed that there was a significant difference in cumulative incidence of overall survival between CHM and non-CHM users (Figure 2; *p* = 0.0025, log-rank test). The results show that the cumulative incidence of overall survival was significantly higher in the CHM users than in non-users.

### CHM Prescription Pattern in Patients With Pemphigus in Taiwan

The most commonly prescribed herbal formulas and single herbs by traditional Chinese Medicine (TCM) doctors in Taiwan for the treatment of patients with pemphigus are listed in Supplementary Table S1. There were two Chinese herbal formulas and five single herbs, and the composition of each was is presented in Supplementary Table S1. Jia–Wei–Xiao–Yao–San (JWXYS) was the most commonly prescribed herbal formula, while Qi–Ju–Di–Huang–Wan (QJDHW) was second. Dan–Shen (*Salvia miltiorrhiza Bunge*) was the most commonly prescribed single herb, followed by Lian–Qiao (*Forsythia suspensa (Thunb.) Vahl*), Huang–Lian (*Coptis chinensis Franch.*), Jin–Yin–Hua (*Lonicera hypoglauca Miq.*), and Di–Gu–Pi (*Lycium barbarum L.*).

The top two CHM combinations for patients with pemphigus were analyzed using the association rule method (Table 3). The prescription frequency, support (%), confidence (%), and lift for these 2-CHM combinations were examined. Stronger associations were defined as those having higher values of frequency of prescription, support, confidence, and lift. As shown in Table 3, the most commonly used two CHM combinations were Qi–Ju–Di–Huang–Wan (QJDHW) → Dan–Shen (DanS) (co-prescription frequency: 197, support: 4.30%, confidence: 55.49%, lift: 5.29), followed by Jia–Wei–Xiao–Yao–San (JWXYS) → Dan–Shen (DanS) (second co-prescription frequency: 135, support: 2.95%, confidence: 35.71%, lift: 3.41) and Huang–Lian (HL) → Qi–Ju–Di–Huang–Wan (QJDHW) (third co-prescription frequency: 118, support: 2.58%, confidence: 43.70%, lift: 5.64).

**TABLE 3:** Most commonly used two CHM combinations for patients with pemphigus in Taiwan.

CHM products (LHS, X)	Chinese name		CHM products (RHS, Y)	Chinese name	Frequency of prescriptions of X and Y products	Support (X) (%)	Confidence (X → Y) (%)	Lift
Qi–Ju–Di–Huang–Wan (QJDHW)	杞菊地黃丸	→	Dan–Shen (DanS)	丹參	197	4.30	55.49	5.29
Jia–Wei–Xiao–Yao–San (JWXYS)	加味逍遙散	→	Dan–Shen (DanS)	丹參	135	2.95	35.71	3.41
Huang–Lian (HL)	黃連	→	Qi–Ju–Di–Huang–Wan (QJDHW)	杞菊地黃丸	118	2.58	43.70	5.64
Jin–Yin–Hua (JYH)	金銀花	→	Lian–Qiao (LQ)	連翹	109	2.38	45.04	7.02
Di–Gu–Pi (DGP)	地骨皮	→	Qi–Ju–Di–Huang–Wan (QJDHW)	杞菊地黃丸	108	2.36	53.47	6.90
Qi–Ju–Di–Huang–Wan (QJDHW)	杞菊地黃丸	→	Jia–Wei–Xiao–Yao–San (JWXYS)	加味逍遙散	107	2.34	30.14	3.65
Di–Gu–Pi (DGP)	地骨皮	→	Dan–Shen (DanS)	丹參	95	2.07	47.03	4.49
Huang–Lian (HL)	黃連	→	Dan–Shen (DanS)	丹參	90	1.97	33.33	3.18
Di–Gu–Pi (DGP)	地骨皮	→	Huang–Lian (HL)	黃連	63	1.38	31.19	5.29

CHM, Chinese herbal medicine; LHS, left-hand-side; RHS, right-hand-side.

Notes: Pemphigus (ICD-9-CM-code: 694.4). Support (X) (%) = Frequency of prescriptions of X and Y products/total prescriptions × 100%. Confidence (X → Y) (%) = Frequency of prescriptions of X and Y products/Frequency of prescriptions of X product × 100%. P (Y) (%) = Frequency of prescriptions of Y product/total prescriptions × 100%. Lift = Confidence (X → Y) (%)/P (Y) (%).

**FIGURE 3:**
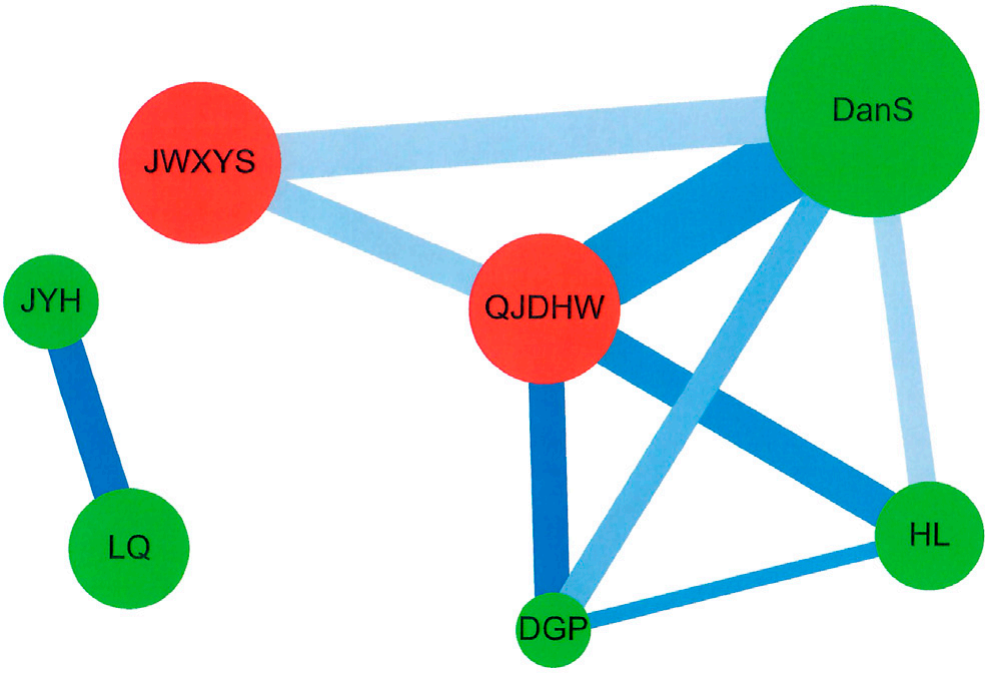
Network analysis for CHM prescription pattern in patients with pemphigus. Abbreviations: CHM, Chinese herbal medicine.

The CHM network and core treatments prescribed for patients with pemphigus were also investigated using network analysis (Figure 3), and CHM constituted networks were constructed. There was one main CHM cluster with Qi–Ju–Di–Huang–Wan (QJDHW), Dan–Shen (DanS), Jia–Wei–Xiao–Yao–San (JWXYS), Huang–Lian (HL), and Di–Gu–Pi (DGP), while the second CHM cluster included Jin–Yin–Hua (JYH) and Lian–Qiao (LQ). Our results show that these seven CHM products were important CHMs for patients with pemphigus in Taiwan.

## Discussion

In this study, we found that for CHM users, the overall survival rate after 6 years decreases to around 90%; for non-CHM user, the overall survival rate decrease to 70%. We also identified two CHM prescription clusters for patients with pemphigus. One main CHM cluster was with Qi–Ju–Di–Huang–Wan (QJDHW), Dan–Shen (DanS), Jia–Wei–Xiao–Yao–San (JWXYS), Huang–Lian (HL), and Di–Gu–Pi (DGP), while the second CHM cluster was with Jin–Yin–Hua (JYH) and Lian–Qiao (LQ). Our results suggested that CHM treatment, especially seven CHM products, was associated with lower risks of overall mortality for patients with pemphigus in Taiwan.

Our results show that patients with pemphigus who were over 60 years old and have a higher CCI index level had higher risks of overall mortality, which were in agreement with previous similar studies (Baican et al., 2015; Jeon et al., 2018). Older age and the presence of more comorbidities were associated with higher risks of overall mortality as observed in Bullous pemphigoid (Jeon et al., 2018). In patients with pemphigus vulgaris (PV), those over 65 years old and have coronary heart disease had higher risks of overall mortality, while in patients with pemphigus foliaceus (PF), those over 65 years old had poor survival (Baican et al., 2015). Furthermore, the leading causes of mortality in pemphigus are infection, especially sepsis and pneumonia (Kridin et al., 2017). Standardized mortality ratios (SMR) for sepsis and pneumonia are 11.57 and 3.64, respectively, in a Taiwan study, and 8.57 and 25.71, respectively, in an Israel study (Huang et al., 2012; Kridin et al., 2017). Cardiovascular disease and chronic renal disease are also associated with higher SMR in both studies.

These studies have prompted the search for complementary therapies to improve the overall survival rate for patients with pemphigus when they get older or develop more comorbidities. Thus, in our study, the effects of CHM therapy on patients with pemphigus in Taiwan was investigated. Our results firstly showed that CHM users had a lower risk of overall mortality than non-CHM users among patients with pemphigus after adjusting for age, gender, prednisolone use, and CCI scores. The cumulative incidence of overall survival was significantly higher in CHM users than in non-users. Our results were in agreement with previous similar studies (Arichi et al., 1979; Zhou et al., 2015b). Chinese medicine can be clinically used and reach a good therapeutic efficacy in combination with glucocorticoids for patients with pemphigus (Arichi et al., 1979; Zhou et al., 2015b). Zhou et al., also reported that glucocorticoid treatment combined with CHM may reduce levels of circulating autoAbs, and decrease adverse events and relapse (Zhou et al., 2015b). Our results also found that there were two CHM clusters for the CHM users. There was one main CHM cluster with Qi–Ju–Di–Huang–Wan (QJDHW), Dan–Shen (DanS; *Radix salviae Miltiorrhizae*; *Salvia miltiorrhiza Bunge*), Jia–Wei–Xiao–Yao–San (JWXYS), Huang–Lian (HL; *Rhizoma coptidis*; *Coptis chinensis Franch*.) and Di–Gu–Pi (DGP; *Cortex lycii*; *Lycium barbarum L.*), while the second CHM cluster included Jin–Yin–Hua (JYH; *Flos lonicerae*; *Lonicera japonica Thunb.*) and Lian–Qiao (LQ; *Fructus forsythiae*; *Forsythia suspensa (Thunb.) Vahl*).

Pathogenic IgG autoAbs-induced signaling is responsible for the intraepidermal blistering observed in pemphigus (Buonavoglia et al., 2019; Didona et al., 2019). This involves the occurrence of Bruton’s tyrosine kinase (BTK) activation (Corneth et al., 2016; Crofford et al., 2016), protein kinase C activation (Osada et al., 1997), sarcoma-associated kinase (Src) activity (Cirillo et al., 2014), Ca^2+^ influx (Seishima et al., 1995), epidermal growth factor receptor binding (Bektas et al., 2013), and most importantly, p38 mitogen-activated protein kinase (MAPK) signaling (Berkowitz et al., 2005; Mavropoulos et al., 2013; Cipolla et al., 2017; Walter et al., 2017). Circulating IgG autoAbs activate the phosphorylation of p38 MAPK. The phosphorylated p38 MAPK then activates heat shock protein 27 (Hsp27) phosphorylation, which finally leads to reorganization of cytoskeleton actin filaments and induced acantholysis (Berkowitz et al., 2005). Phosphorylated p38 MAPK also induces the activation of Nuclear factor-κB (NF-Kb) and cAMP-response element binding (CREB) proteins and leads to increased inflammatory IFN, IL-6, IL-8 cytokine and furin protein expressions, which facilitate Dsg1 and Dsg3 protein maturations (Kumar et al., 2010; Zhou et al., 2014; Cipolla et al., 2017). Therefore, one of the possible therapeutic approaches for pemphigus is to target p38 MAPK signaling. Studies have shown that our seven Chinese herbal products, including Jia–Wei–Xiao–Yao–San (JWXYS), Qi–Ju–Di–Huang–Wan (QJDHW), Dan–Shen (DanS; *Radix salviae Miltiorrhizae*; *Salvia miltiorrhiza Bunge*), Lian–Qiao (LQ; *Fructus dorsythiae*; *Forsythia suspensa (Thunb.) Vahl*), Huang–Lian (HL; *Rhizoma coptidis*; *Coptis chinensis Franch*.), Jin–Yin–Hua (JYH; *Flos lonicerae*; *Lonicera japonica Thunb.* and Di–Gu–Pi (DGP; *Cortex lycii*; *Lycium barbarum L.*), all reduced the phosphorylation of p38 MAPK protein (Choi et al., 2013; Song et al., 2014; Kao et al., 2015; Chao et al., 2016; Tsai et al., 2017b).

The herbal biomarkers of Jia–Wei–Xiao–Yao–San include saikosaponin A, saikosaponin D, ferulic acid and paeoniflorin (Lu et al., 2013). All of these components lead to inhibition of both of the p38 MAPK and NF-kB pathways (Guo et al., 2012; Chen et al., 2013; Zhu et al., 2013; Lin et al., 2015a; Ma et al., 2015; Zhou et al., 2015a; Lin et al., 2016; Cai et al., 2017; Koshiguchi et al., 2017; Zhang et al., 2017a; Yin et al., 2019). The herbal biomarkers of Qi–Ju–Di–Huang–Wan are alisol B, pachymic acid, alisol C, rutin, luteolin, cornuside, paeoniflorin, catapol and diosgenin (Jarouche et al., 2019). Among them, paeoniflorin has also been detected in Jia–Wei–Xiao–Yao–San. Furthermore, alisol B, pachymic acid, rutin, luteolin, cornuside, catalpol, and diosgenin inhibit the activation of both the p38 MAPK and NF-kB pathways (Ling et al., 2011; Li et al., 2015; Li et al., 2016; Zhang et al., 2017b; Cai et al., 2018; Song et al., 2018; Wang et al., 2019; Che et al., 2020; Chen et al., 2020). Natural compounds including salvianolic acid B, caffeic acid, and tanshinone IIA have been detected in Dan–Shen (*Radix salviae Miltiorrhizae*; *Salvia miltiorrhiza Bunge*) (Pang et al., 2016). These three natural compounds inhibit both p38 MAPK and NF-kB pathways (Fang et al., 2018; Li et al., 2019; Liu et al., 2019). Natural compounds including kaempferol, forsythoside A, phillyrin, rutin and phillygenin have been detected in Lian–Qiao (*Fructus Forsythiae*; *Forsythia suspensa (Thunb.) Vahl*) (Wang et al., 2018), with rutin also detected in Qi–Ju–Di–Huang–Wan. Among these natural compounds, kaempferol and forsythoside A suppress both the p38 MAPK and NF-kB pathways (Liu et al., 2017; Hwang et al., 2019). Natural compounds including coptisine, berberine, and palmatine have been detected in Huang–Lian (*Rhizoma Coptidis*; *Coptis chinensis Franch.*) (Tian et al., 2017), with coptisine and berberine exhibiting anti-inflammatory activity through inhibition of both the p38 MAPK and NF-kB pathways (Chen et al., 2017; Zhao et al., 2019). Natural compounds including kaempferol, ursolic acid, and rutin have been detected in Jin–Yin–Hua (*Flos Lonicerae*; *Lonicera japonica Thunb.*) (Cai et al., 2019), with kaempferol and rutin also detected in Lian–Qiao and Qi–Ju–Di–Huang–Wan. Ursolic acid is able to suppress both the p38 MAPK and NF-kB pathways (Ma et al., 2014). Natural compounds including kukoamines A and B have been detected in Di–Gu–Pi (*Cortex Lycii*; *Lycium barbarum L.*) (Li et al., 2017), with kukoamine B found to suppress the p38 MAPK pathway (Hu et al., 2015). These results suggest that Chinese herbs and related natural compounds may be potential candidates for the treatment of pemphigus pathogenesis via inhibiting both the p38 MAPK and NF-kB pathways.

The main limitation was the lacks of laboratory tests, education, occupation, and lifestyle demographics of these patients. However, our results showed that CHM may be associated with a reduced risk of overall mortality, and these CHMs may be useful for future investigations in randomized controlled trials (RCT) and functional studies. Large-scale RCTs may be performed to determine their relative effectiveness and safety, and to evaluate their potential interactions during regular treatments in patients with pemphigus.

## Conclusion

Patients with pemphigus who used CHM as an adjunctive therapy had better survival rates. Among the CHMs explored, Qi–Ju–Di–Huang–Wan (QJDHW), Dan–Shen (DanS; *Radix salviae Miltiorrhizae*; *Salvia miltiorrhiza Bunge*), Jia–Wei–Xiao–Yao–San (JWXYS), Lian–Qiao (LQ; *Fructus forsythiae*; *Forsythia suspensa (Thunb.) Vahl*), Huang–Lian (HL; *Rhizoma coptidis*; *Coptis chinensis Franch*.), Jin–Yin–Hua (JYH; *Flos lonicerae*; *Lonicera hypoglauca Miq.*), and Di–Gu–Pi (DGP; *Cortex lycii*; *Lycium barbarum L.*) were found to be the most effective for pemphigus. Further studies should be performed to optimize the safety and efficacy of CHMs for patients with pemphigus, as well as for functionally investigating other potential compounds for pemphigus treatment.

## Data Availability Statement

The original contributions presented in the study are included in the article/Supplementary Material, further inquiries can be directed to the corresponding authors.

## Ethics Statement

This study was approved by the Research Ethics Committee of the Taiwan National Health Research Institute and the Institutional Review Board of the China Medical University Hospital (ethics approval number: CMUH107-REC3-074 (CR1)). Written informed consent for participation was not required for this study in accordance with the national legislation and the institutional requirements.

## Author Contributions

Y-JL, P-YW, T-ML, and S-IC wrote the manuscript and interpreted the data. C-JC, J-SC, M-KL, Y-CW, T-HL, C-CL, S-MH, and Y-NL collected, assembled, and analyzed the data. F-JT and Y-JL provided study materials. W-ML and Y-JL designed, conceived the study, and amended the manuscript.

## Funding

This study was supported by grants from China Medical University (CMU108-MF-32, CMU108-S-15, and CMU108-S-17), China Medical University Hospital (DMR-109-145, DMR-109-188, and DMR-109-192), and the Ministry of Science and Technology, Taiwan (MOST 106-2320-B-039 -017 -MY3, MOST 108-2314-B-039-044-MY3, and MOST 109-2320-B-039-035-MY3).

## Conflict of Interest

The authors declare that the research was conducted in the absence of any commercial or financial relationships that could be construed as a potential conflict of interest.
