# Supplementary material for: Complementary Chinese Herbal Medicine Therapy Improves Survival in Patients With Pemphigus: A Retrospective Study From a Taiwan-Based Registry
Source: Front Pharmacol. 2020 Dec 9;11:594486. doi: 10.3389/fphar.2020.594486 (PMC7756119; doi:10.3389/fphar.2020.594486)
Supplement: Supplementary file 1 [file datasheet1.docx]

**Supplementary Information**

Supplementary Text

Figures S1-S7

Table S1

**Supplementary Text**

**Figure S1.** LC-MS/MS analysis of active component standards and the extracts of Jia-Wei-Xiao-Yao-San (JWXYS). LC-MS/MS analysis of active component standards and the extracts of glycyrrhizin. (B) Base peak chromatogram (BPC) and EIC of the extracts of JWXYS.

**Figure S2.** Certificate of analysis for Qi-Ju-Di-Huang-Wan (QJDHW).

**Figure S3.** LC-MS/MS analysis of active component standards and the extracts of Dan-Shen (DanS). LC-MS/MS analysis of active component standards and the extracts of tanshinone I and salvianolic acid B. (B) Base peak chromatogram (BPC) and EIC of the extracts of DanS.

**Figure S4.** LC-MS/MS analysis of active component standards and the extracts of Lian-Qiao (LQ). LC-MS/MS analysis of active component standards and the extracts of ursolic acid. (B) Base peak chromatogram (BPC) and EIC of the extracts of LQ.

**Figure S5.** LC-MS/MS analysis of active component standards and the extracts of Huang-Lian (HL). LC-MS/MS analysis of active component standards and the extracts of berberine and coptisine. (B) Base peak chromatogram (BPC) and EIC of the extracts of HL.

**Figure S6.** LC-MS/MS analysis of active component standards and the extracts of Jin-Yin-Hua (JYH). LC-MS/MS analysis of active component standards and the extracts of chlorogenic acid and loganin. (B) Base peak chromatogram (BPC) and EIC of the extracts of JYH.

**Figure S7.** LC-MS/MS analysis of active component standards and the extracts of Di-Gu-Pi (DGP). LC-MS/MS analysis of active component standards and the extracts of linoleic acid and scopoletin. (B) Base peak chromatogram (BPC) and EIC of the extracts of DGP.

**Table S1.** Composition of the most commonly used herbal formulas and single herbs for patients with pemphigus in Taiwan.

**Fig. S1 A**

**Standard reference compounds for Jia-Wei-Xiao-Yao-San (JWXYS)**

**
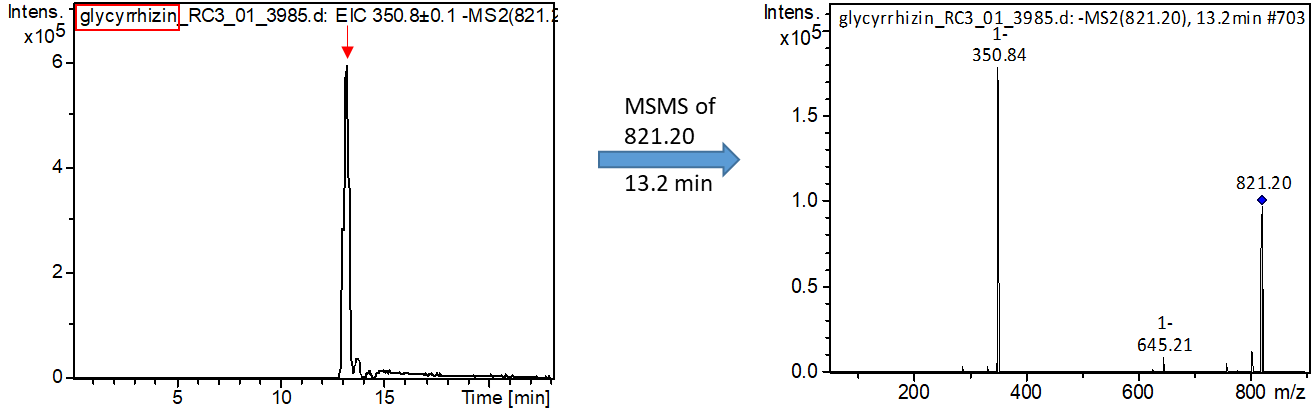
**

**Fig. S1 B**

**Jia-Wei-Xiao-Yao-San (JWXYS) extract**

**
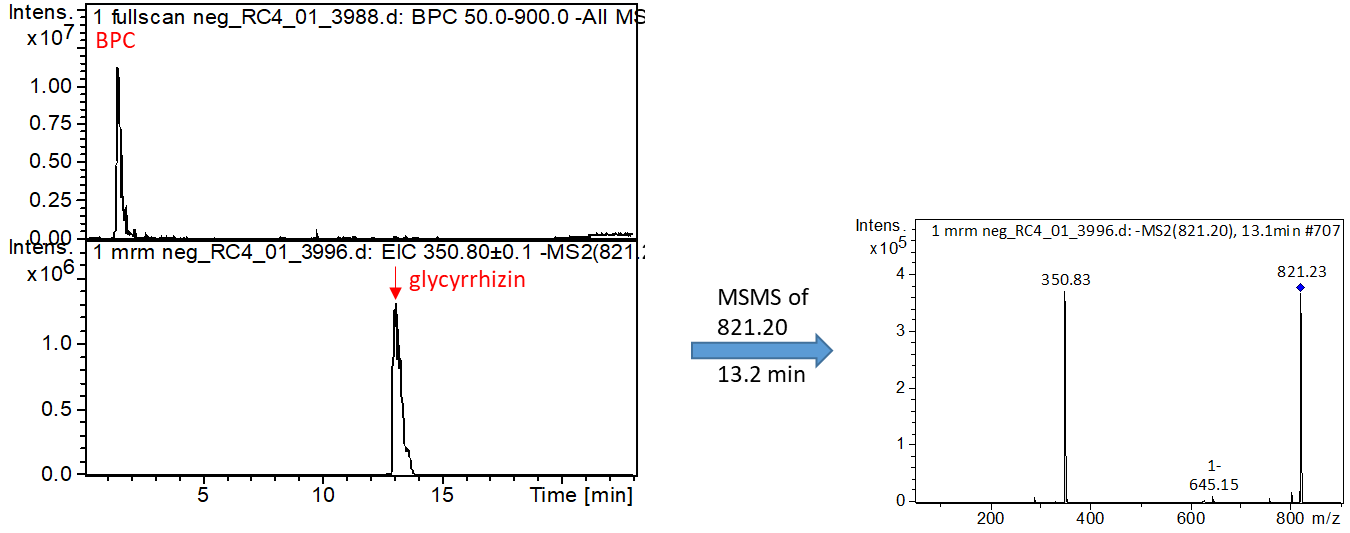
**

**Fig. S2**

**Qi-Ju-Di-Huang-Wan (QJDHW) 杞菊地黃丸**

**Qi-Ju-Di-Huang-Wan (QJDHW) 杞菊地黃丸**

**Fig. S2**

**Qi-Ju-Di-Huang-Wan (QJDHW)** **杞菊地黃丸**

**Fig. S2**

**Qi-Ju-Di-Huang-Wan (QJDHW) 杞菊地黃丸**

**Fig. S3 A**

**Standard reference compounds for Dan-Shen (DanS)**


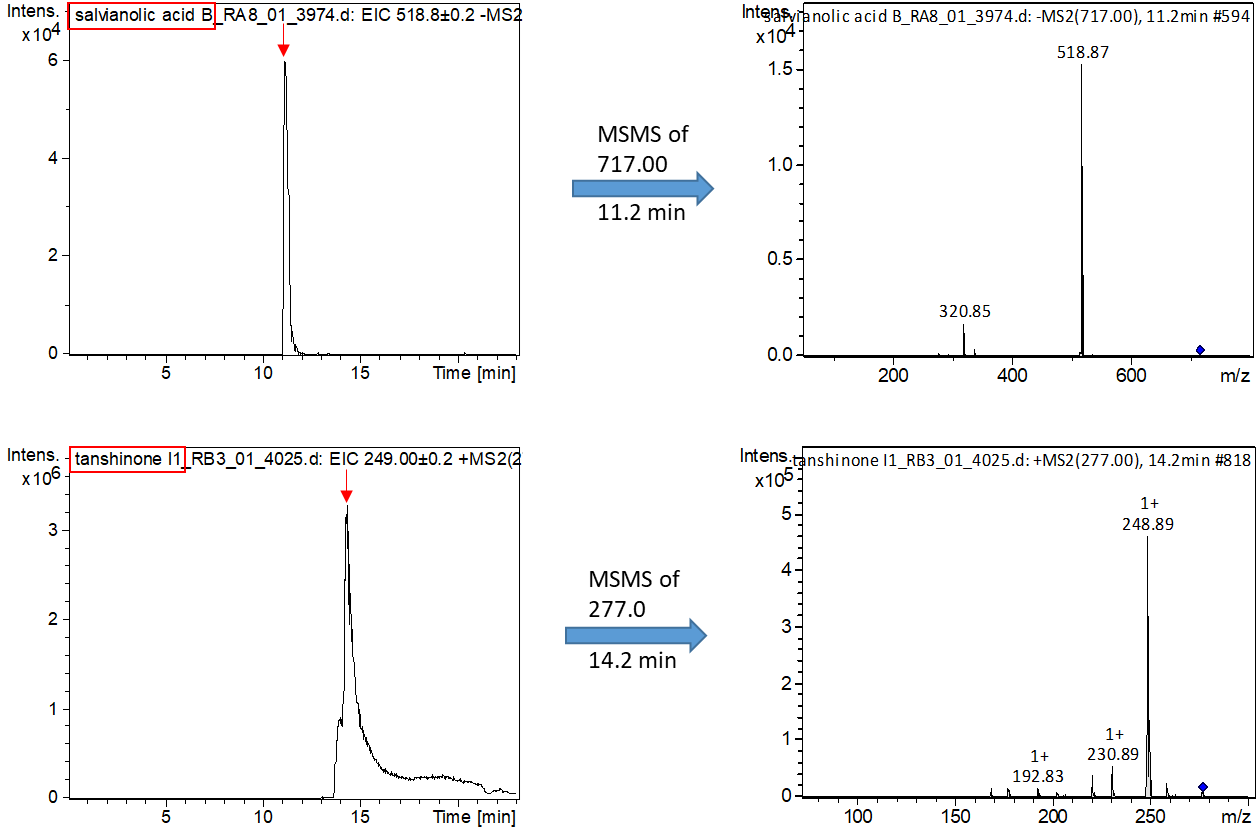


**Fig. S3 B**

**Dan-Shen (DanS) extract**


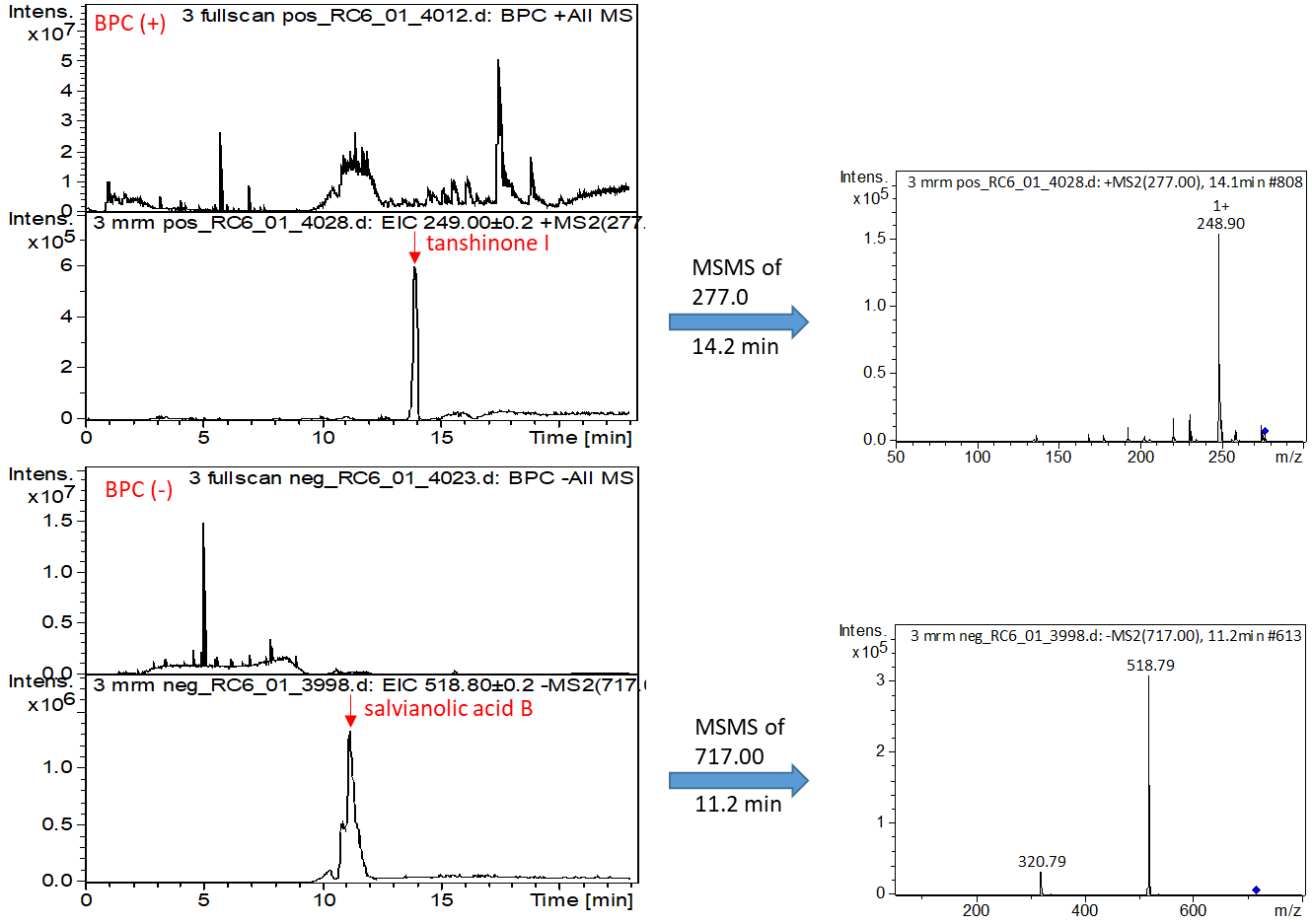


**Fig. S4 A**

**Standard reference compounds for Lian-Qiao (LQ)**


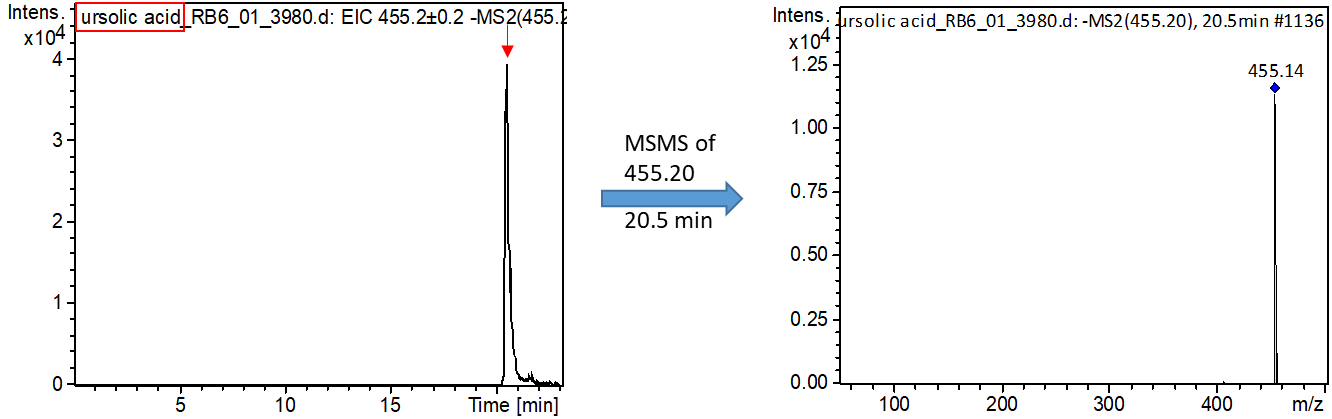


**Fig. S4 B**

**Lian-Qiao (LQ) extract**


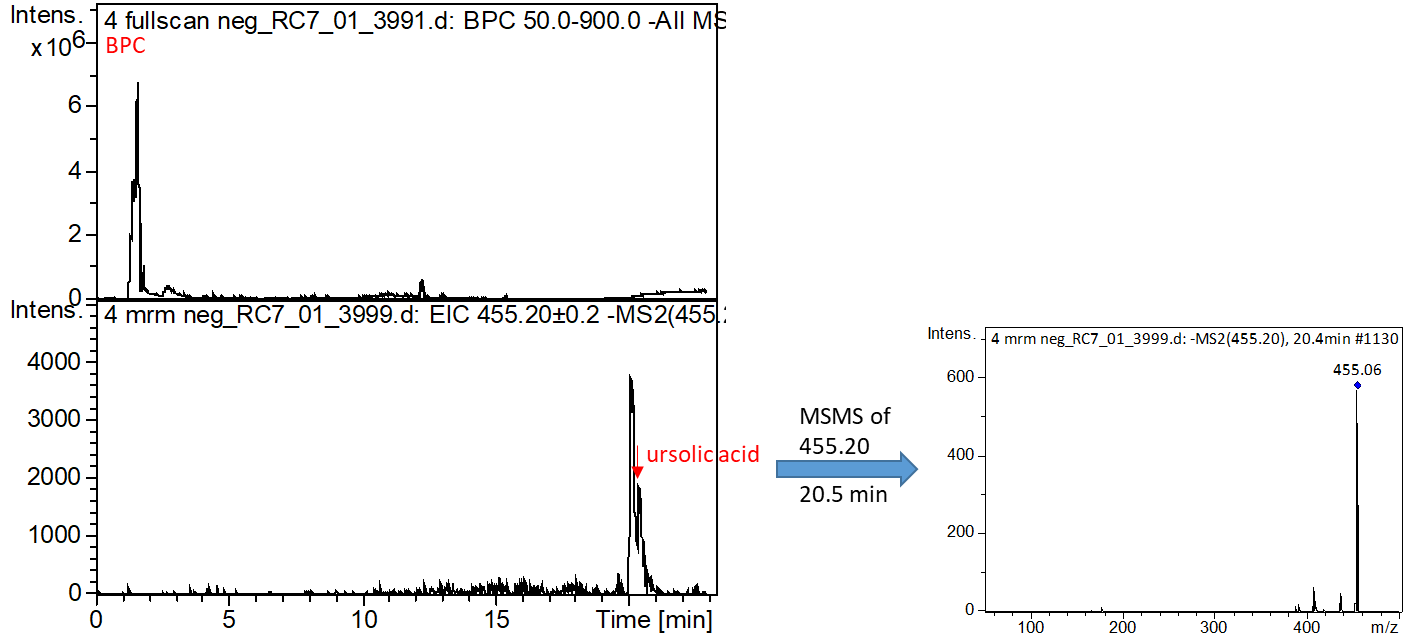


**Fig. S5 A**

**Standard reference compounds for Huang-Lian (HL)**

**
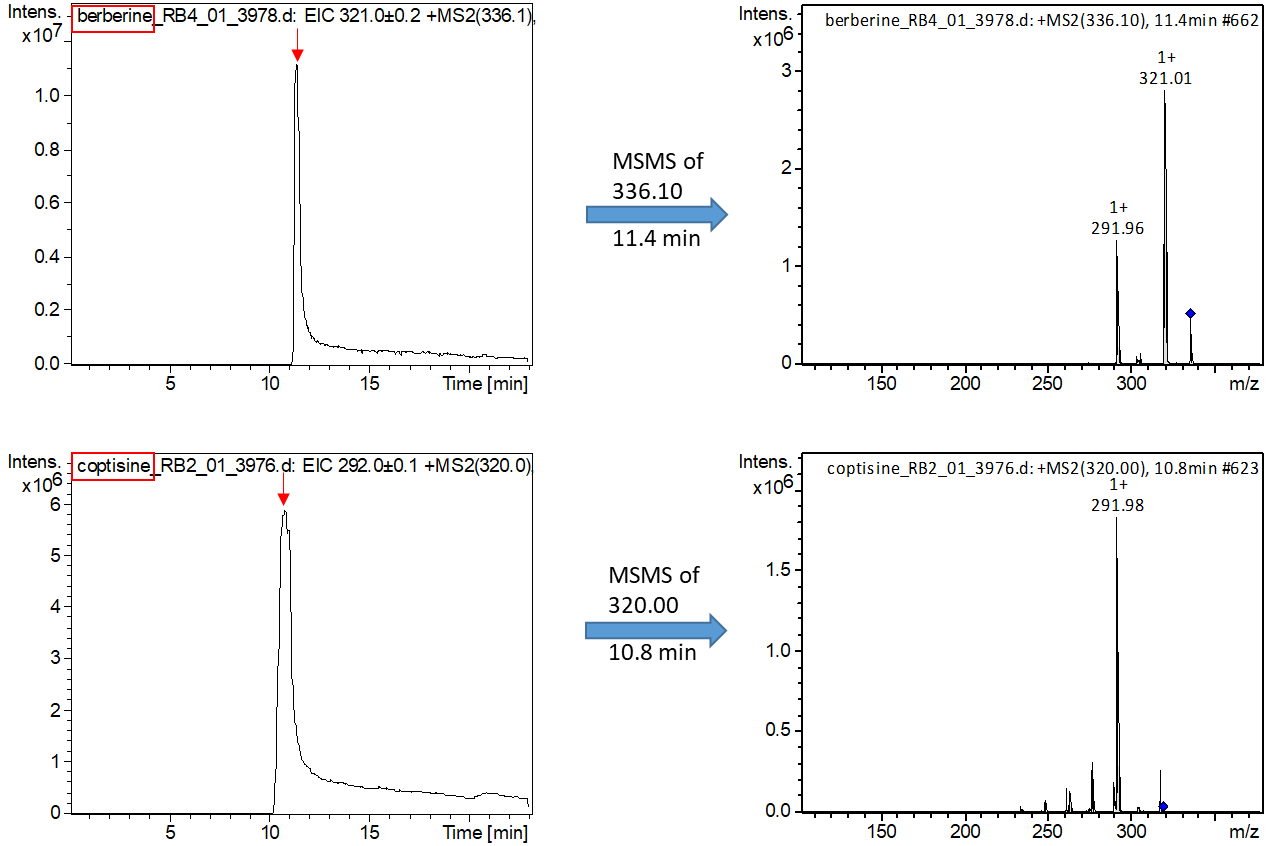
**

**Fig. S5 B**

**Huang-Lian (HL) extract**

**
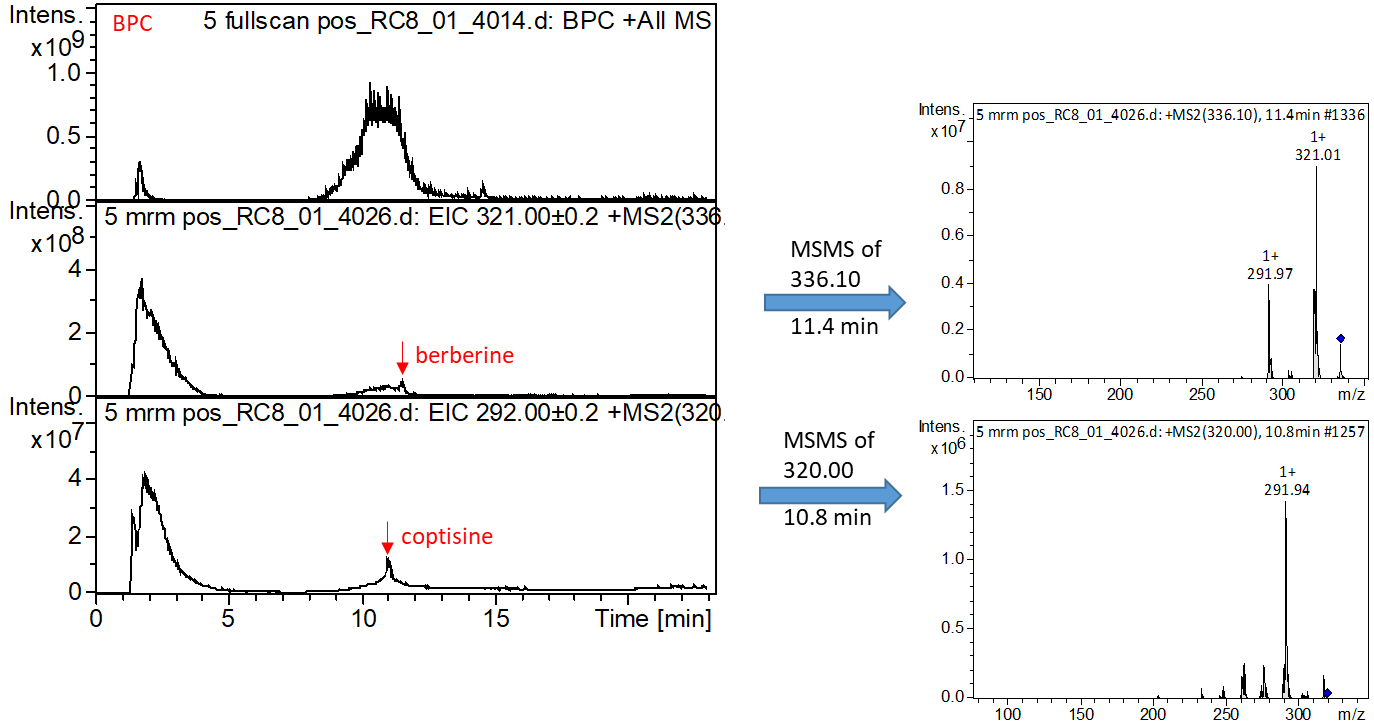
**

**Fig. S6 A**

**Standard reference compounds for Jin-Yin-Hua (JYH)**

**
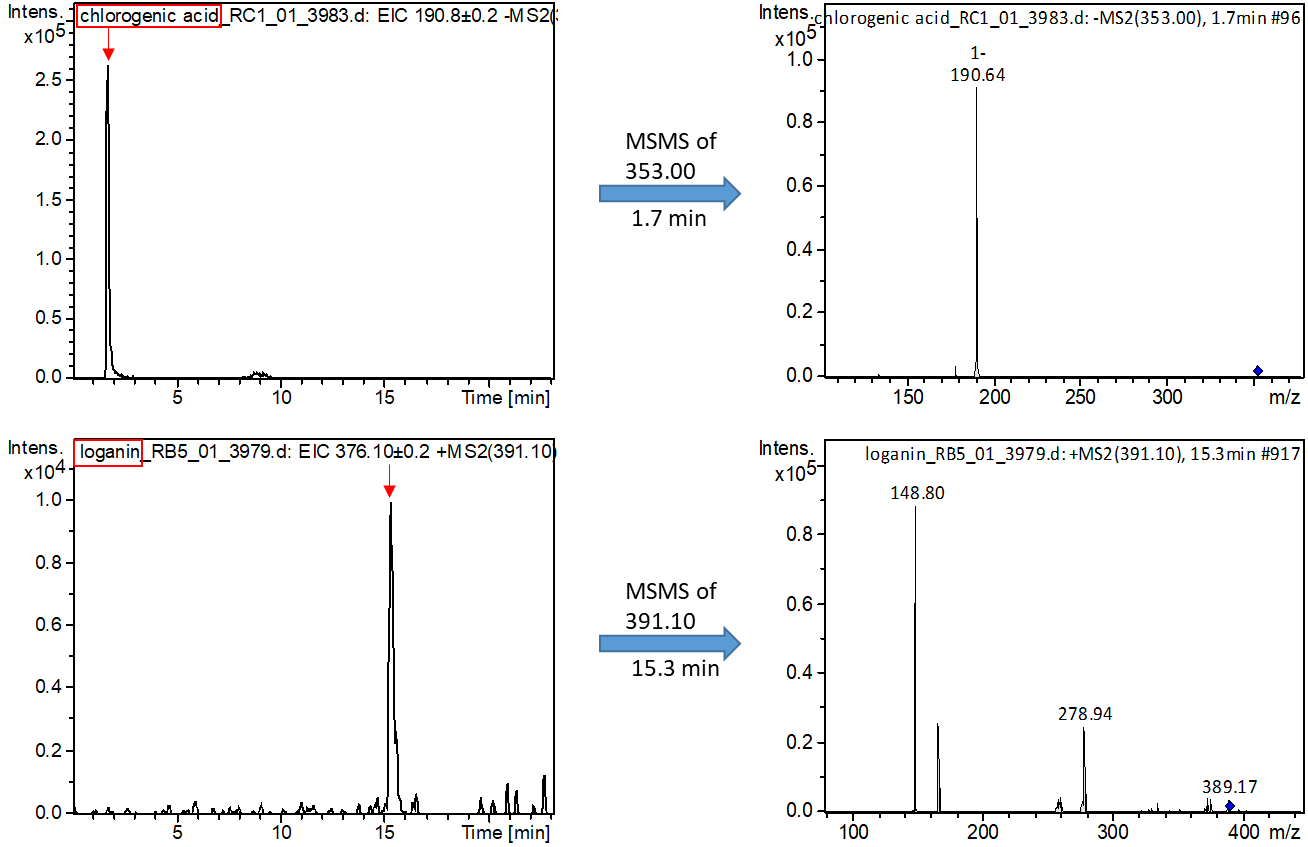
**

**Fig. S6 B**

**Jin-Yin-Hua (JYH) extract**

**
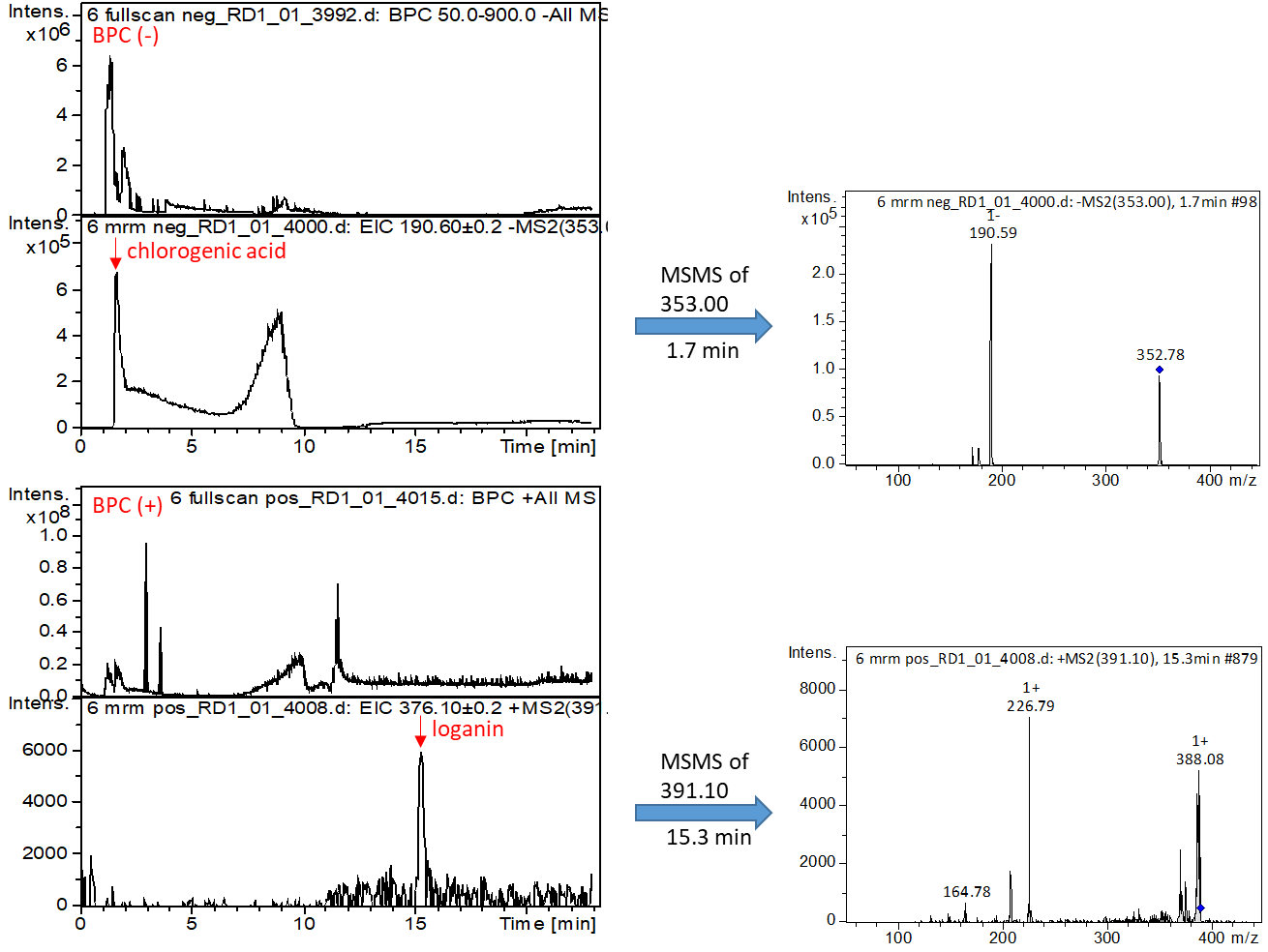
**

**Fig. S7 A**

**Standard reference compounds for Di-Gu-Pi (DGP)**

**
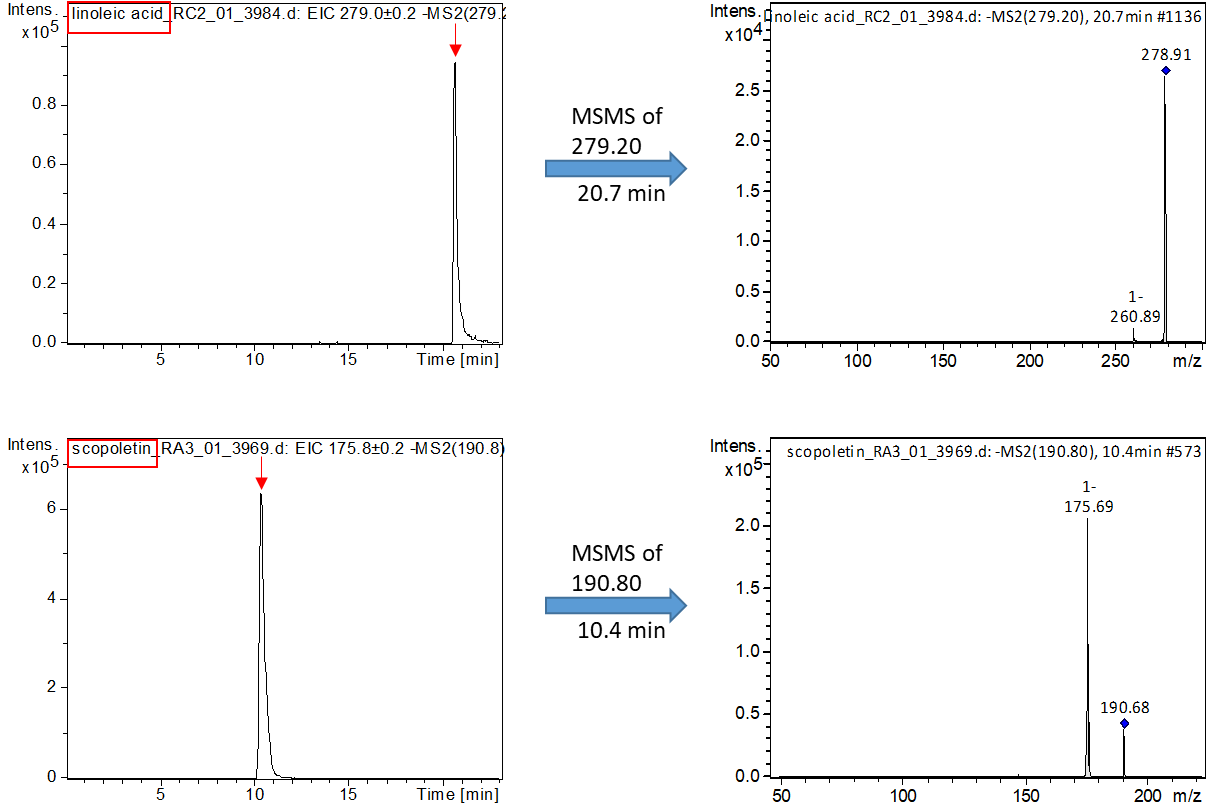
**

**Fig. S7 B**

**Di-Gu-Pi (DGP) extract**

**
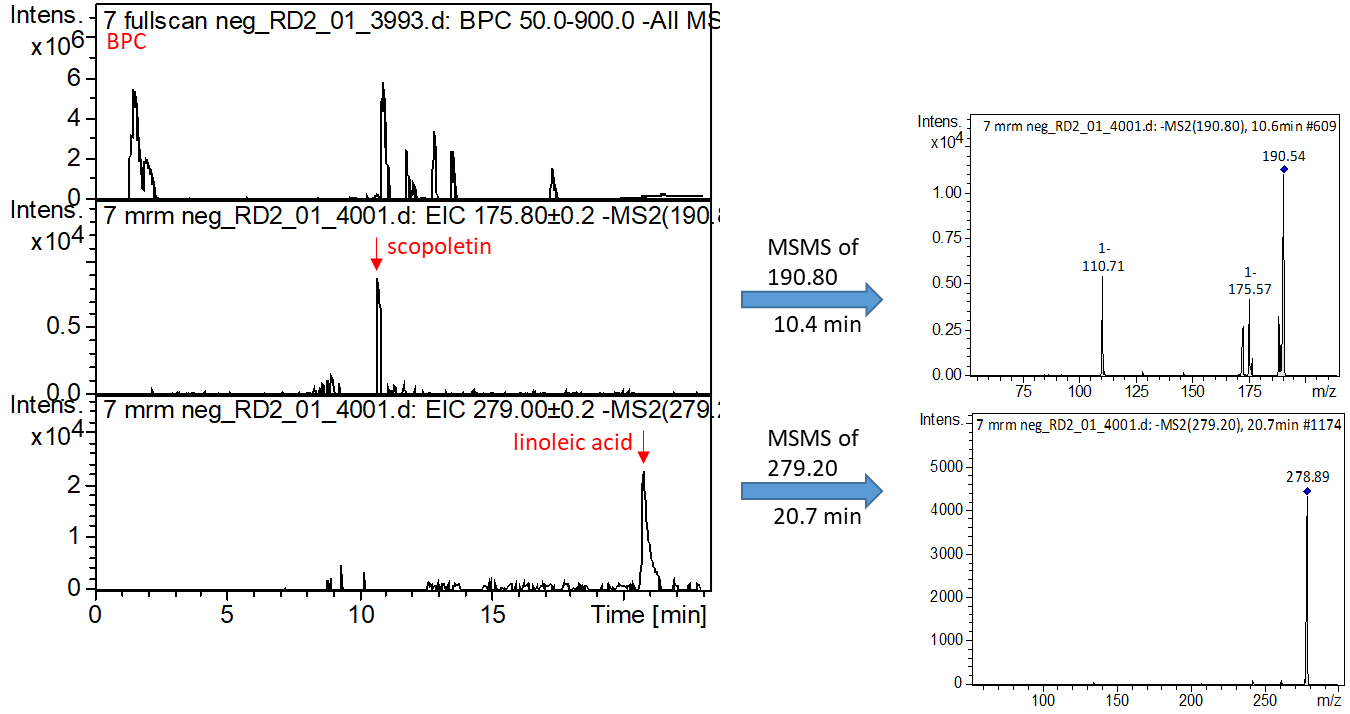
**

| **TABLE S1 \|** Composition of the most commonly used herbal formulas and single herbs for patients with pemphigus in Taiwan. | | | | | | | | |
| --- | --- | --- | --- | --- | --- | --- | --- | --- |
| **Formulas** | **Chinese name** | **Number of herbs** | **Composition (Pin-yin name (latin name; botanical plant name))** | **Frequency of prescriptions** | **Person-year** | **Percentage of usage person** | **Avg. drug dose per day (g)** | **Average duration for prescription (days)** |
| **Total** |  |  |  | **4580** | **906.2** | **100.0** | **14.1** | **8.9** |
| **Herbal formula (Pin-yin name)** |  |  |  | **4476** | **906.2** | **100.0** | **10.0** | **9.0** |
| Jia-Wei-Xiao-Yao-San (JWXYS) | 加味逍遙散 | 10 | **Dang-Gui** (*Radix Angelicae Sinensi*; *Angelica sinensis (Oliv.) Diels*), **Bai-Shao** (*Radix Paeoniae Alba*; *Paeonia lactiflora Pall.*), **Fu-Ling** (*Poria*; *Wolfiporia extensa (Peck) Ginns*), **Bai-Zhu** (*Rhizoma Atractylodis Macrocephalae*; *Atractylodes macrocephala Koidz.*), **Chai-Hu** (*Radix Bupleuri*; *Bupleurum falcatum L.*), **Mu-Dan-Pi** (*Cortex Moutan*; *Moutan officinalis (L.) Lindl. & Paxton*), **Zhi-Zi** (*Fructus Gardeniae*; *Gardenia jasminoides J.Ellis*), **Gan-Cao** (*Radix Glycyrrhizae Preparata*; *Glycyrrhiza uralensis Fisch.*), **Bo-He** (*Herba Menthae Haplocalycis*; *Mentha arvensis L.*), **Sheng-Jiang** (*Rhizoma Zingiberis Recens*; *Zingiber officinale Roscoe*) | 378 | 380.8 | 36.6 | 4.3 | 9.0 |
| Qi-Ju-Di-Huang-Wan (QJDHW) | 杞菊地黃丸 | 8 | **Gou-Qi (***Fructus Lycii***;** *Lycium barbarum L.***), Ju-Hua (** *Flos Chrysanthemi* **;** *Chrysanthemum indicum L.* **), Shu-Di-Huang (***Radix Rehmanniae Preparata***;** *Rehmannia glutinosa (Gaertn.) DC.***),** **Shan-Zhu-Yu (***Fructus Corni***;** *Cornus officinalis Siebold & Zucc.***), Shan-Yao (***Rhizoma Dioscoreae***;** *Dioscorea oppositifolia L.***), Ze-Xie (***Rhizoma Alismatis;* *Alisma plantago-aquatica L.***), Fu-Ling** (*Poria*; *Wolfiporia extensa (Peck) Ginns*), **Mu-Dan-Pi (***Cortex Moutan***;** *Moutan officinalis (L.) Lindl. & Paxton***)** | 355 | 221.8 | 20.6 | 5.1 | 12.9 |
| **Single herbs (Pin-yin name)** |  |  |  | **4131** | **899.0** | **98.5** | **4.7** | **9.0** |
| Dan-Shen (DanS) | 丹參 | 1 | **Dan-Shen** (*Radix Salviae Miltiorrhizae ; Salvia miltiorrhiza Bunge*) | 480 | 348.9 | 33.6 | 1.3 | 10.9 |
| Lian-Qiao (LQ) | 連翹 | 1 | **Lian-Qiao** (*Fructus Forsythiae; Forsythia suspensa (Thunb.) Vahl*) | 294 | 391.9 | 39.7 | 1.2 | 8.6 |
| Huang-Lian (HL) | 黃連 | 1 | **Huang-Lian** (*Rhizoma Coptidis*; *Coptis chinensis Franch.*) | 270 | 308.6 | 30.5 | 1.4 | 11.5 |
| Jin-Yin-Hua (JYH) | 金銀花 | 1 | **Jin-Yin-Hua** (*Flos Lonicerae*; *Lonicera hypoglauca Miq.*) | 242 | 310.7 | 33.6 | 1.3 | 7.8 |
| Di-Gu-Pi (DGP) | 地骨皮 | 1 | **Di-Gu-Pi** (*Cortex Lycii*; *Lycium barbarum L.*) | 202 | 250.2 | 22.1 | 1.7 | 13.9 |
| *Sorted by frequency of prescriptions. | | | | | | | | |
| Information are obtained from the websites (http://www.americandragon.com/index.htm; http://old.tcmwiki.com/; http://www.shen-nong.com/eng/front/index.html; http://www.ipni.org/; http://www.theplantlist.org/). | | | | | | | | |
